# Concerns, perceived need and competing priorities: a qualitative exploration of decision-making and non-participation in a population-based flexible sigmoidoscopy screening programme to prevent colorectal cancer

**DOI:** 10.1136/bmjopen-2016-012304

**Published:** 2016-11-11

**Authors:** N Hall, L Birt, C J Rees, F M Walter, S Elliot, M Ritchie, D Weller, G Rubin

**Affiliations:** 1 School of Pharmacy, Medicine and Health, Durham University, Stockton on Tees, UK; 2 Faculty of Applied Sciences, University of Sunderland, Sunderland, UK; 3 Department of Public Health and Primary Care, University of Cambridge, Cambridge, UK; 4 University of East Anglia, Norwich, UK; 5 South Tyneside NHS Foundation Trust, South Shields, UK; 6 South of Tyne NHS Bowel Cancer Screening Centre, Queen Elizabeth Hospital, Gateshead, UK; 7 Lay Member of Steering Committee, Gateshead, UK; 8 Cancer Research Centre, Edinburgh University, Edinburgh, UK

**Keywords:** screening, colorectal, cancer, flexible-sigmoidoscopy, non-participation, QUALITATIVE RESEARCH

## Abstract

**Objective:**

Optimising uptake of colorectal cancer (CRC) screening is important to achieve projected health outcomes. Population-based screening by flexible sigmoidoscopy (FS) was introduced in England in 2013 (NHS Bowel scope screening). Little is known about reactions to the invitation to participate in FS screening, as offered within the context of the Bowel scope programme. We aimed to investigate responses to the screening invitation to inform understanding of decision-making, particularly in relation to non-participation in screening.

**Design:**

Qualitative analysis of semistructured in-depth interviews and written accounts.

**Participants and setting:**

People from 31 general practices in the North East and East of England invited to attend FS screening as part of NHS Bowel scope screening programme were sent invitations to take part in the study. We purposively sampled interviewees to ensure a range of accounts in terms of beliefs, screening attendance, sex and geographical location.

**Results:**

20 screeners and 25 non-screeners were interviewed. Written responses describing reasons for, and circumstances surrounding, non-participation from a further 28 non-screeners were included in the analysis. Thematic analysis identified a range of reactions to the screening invitation, decision-making processes and barriers to participation. These include a perceived or actual lack of need; inability to attend; anxiety and fear about bowel preparation, procedures or hospital; inability or reluctance to self-administer an enema; beliefs about low susceptibility to bowel cancer or treatment and understanding of harm and benefits. The strength, rather than presence, of concerns about the test and perceived need for reassurance were important in the decision to participate for screeners and non-screeners. Decision-making occurs within the context of previous experiences and day-to-day life.

**Conclusions:**

Understanding the reasons for non-participation in FS screening can help inform strategies to improve uptake and may be transferable to other screening programmes.

**Strengths and limitations of this study**

- Qualitative methods used within this study allowed an in-depth exploration of the contexts, decision-making processes and emotional reactions rooted within the reasons provided for non-participation in colorectal cancer screening.
- Our recruitment strategy allowed for anticipated difficulties in recruiting non-screeners, however, the overall response to our study invitation remained low.
- Purposive sampling ensured that we were able to include accounts from a full range of participants in terms of their beliefs, decision-making and attendance.
- Our sampling allowed us to compare a diverse range of accounts from screeners and non-screeners within and across research sites and screening centres, including participants living in the most deprived areas within the UK.
- Our sample did not include enough respondents from ethnic minority groups to draw conclusions about more specific cultural influences.

## Introduction

Colorectal cancer (CRC) screening is important in reducing CRC-related mortality.^1–3^ In England, a flexible sigmoidoscopy (FS) test at 55 years has been added to the existing faecal occult blood testing (FOBT) population-based CRC screening programme offered between 60 and 74 years.^4^ Since 2013, the FS programme, termed Bowel scope, has been progressively implemented across the UK through regional bowel screening centres. Each centre covers a geographical population which is served by a number of screening sites (endoscopy units). Each centre is expected to have at least one site offering FS screening by the end of 2016, with complete coverage of the English population expected around 3 years after that. The primary purpose of FS screening is to prevent CRC by identifying and removing adenomas before they develop malignant changes. It has been shown to reduce CRC mortality and incidence in the UK,^1^ ^5^ Europe and the USA.^6^ The effectiveness of any population-based screening programme is reliant on high uptake. At 43.1%, CRC screening uptake is lower than breast or cervical cancer screening (even among women),^7^ and uptake for FS is lower than that for FOBT.^4^ ^8^ Understanding the influences on decision-making and non-participation in FS screening is therefore important to help optimise projected gains in mortality and reduce health inequalities.

A number of sociodemographic, ethnic and sociological influences on FOBT screening participation have been identified.^9–14^ Intervention studies incorporating factors such as general practitioner (GP) endorsement,^15^ reminders and social networks have shown these can have a positive effect on uptake. However, the evidence is inconsistent,^16^ ^17^ and effectiveness is likely to be, in part, influenced by the healthcare context in which the intervention is based. The dynamics of decision-making for FS screening may be quite different, with its high technology, specialist-based approach, a less proactive role required for participants and a different method of invitation. Qualitative research among participants in the UK FS Trial^1^ has identified that most of the effects of demographic and health variables on interest in participation are mediated by sociocognitive variables,^18^ although actual uptake among interested participants is influenced more directly by demographics, health and stress.^19^ Non-participation is also reported to be influenced by avoidant attitudes towards screening, other health beliefs,^20^ ^21^ fear^22^ and deprivation.^23^ This research has been undertaken with people offered screening within a research trial context or has focused on intention rather than actual screening behaviour. A quantitative analysis of screening uptake in the first 14 months of the English Bowel scope screening programme identified independent effects of deprivation, gender and screening centre on screening participation.^7^ We sought to gain a more thorough understanding of the influences on screening participation by conducting an in-depth exploration of the responses of members of the public to their invitation to the Bowel scope screening programme. By taking this approach, we obtained their reflections on their actual decision-making and experiences, their awareness and understanding of CRC and the contexts surrounding reasons for non-participation.

## Methods

### Setting

The study took place across two research sites (the North East England and East of England), chosen because of their diverse deprivation profiles and their location within areas covered by two of the first English pilot NHS Bowel scope screening centres. The study was provided a favourable ethical opinion by the NHS Bromley NRES Committee (14/LO/0207).

### Participant recruitment

For each general practice included in the screening programme, the NHS CRC screening hubs generate a letter inviting patients aged 55 years to attend for FS screening at their local Bowel scope screening centre. Thirty-one eligible general practices (18 North East England; 13 East of England) agreed to mail study information explaining the aims of the study and a study sampling questionnaire to all patients invited for FS screening within the previous 6 months. The Bowel scope screening invitation process takes 8 weeks from initial contact to appointment date, and no study information was sent to patients during this time to avoid influencing their decision-making. Recruitment took place between March and December 2014.

Participants were asked to return the sampling questionnaire directly to the study researchers indicating whether they wished to be contacted for a face-to-face interview. The sampling questionnaire gathered information on gender, ethnicity, screening attendance and a series of items to assess attitudes towards cancer, concerns about the FS test and current bowel symptoms. This information was used to purposively sample participants for interview, helping to ensure we interviewed people who had attended FS screening (screeners) and those who had not attended (non-screeners), including people with a range of attitudes, beliefs and reasons for attending and not attending screening.

Study information was initially sent to 623 eligible patients. There were lower rates of questionnaire return among the non-screeners (36%) than the screeners (61%). To adhere to the principles of qualitative purposive sampling,^24^ after 5 months of recruitment we only sent recruitment material to non-screeners, thereby helping to ensure we recruited from this typically hard to reach group. At this stage, study information was posted to a further 552 non-screeners in phases until we had interviewed 25 non-screeners and 20 screeners and no new themes were emerging from the participants’ accounts.

The sampling questionnaire included an open question inviting written responses for reasons for non-attendance. By interviewing people who had undergone and not undergone screening, we were able to explore similarities and differences in beliefs and decision-making processes.

### Data collection

Semistructured, face-to-face interviews were undertaken in the participant's home by NH and LB, both experienced postdoctoral qualitative researchers. Interviews were preceded by an explanation of the research, a reiteration that the researcher was not a member of the FS screening team or the general practitioner (GP) practice. Written consent was obtained before the interview started. The semistructured interview guide included general open-ended questions on reasons for attending or not-attending screening, concerns about FS screening, understanding of CRC and views on screening within the NHS. Participant-initiated topics were encouraged and pursued during the interviews. Additional interview prompts included knowledge, beliefs and previous experiences of cancer in general and cancer screening more specifically; practicalities, concerns and experiences associated with screening attendance and bowel preparations and participation in other screening programmes. Interviews lasted between 30 min and 50 min and were audio-recorded. All written responses to an open-ended question included within the sampling questionnaire (“please let us know below if there were any other reasons or circumstances which meant you were unable to, or did not wish to, take part in Bowel scope screening”) were recorded for analysis.

### Qualitative analysis

All interviews were transcribed verbatim. Interview transcripts and written responses were analysed using thematic analysis.^25^ After familiarising themselves with the data, NH and LB initially worked independently then collaboratively to develop a data-driven coding framework. Once data were organised into codes, using word processing software, NH and LB searched for patterns and developed early themes, exploring similarities and differences between ‘screeners’ and ‘non-screeners’. These themes were reviewed for credibility by referring back to the empirical literature on screening behaviour. Peer validation was sought through sharing with the study steering group. This group comprised clinicians and academics with expertise in screening research and a service user, with experience of FS screening and an interest in the bowel cancer screening programme.

## Results

We received a total of 214 sampling questionnaires (88 non-screeners; overall response rate 18%), 110 of whom agreed to be contacted for interview (32 non-screeners). Responses to the items on the sampling questionnaire indicated that the majority of screeners and non-screeners held positive beliefs about CRC screening (screeners 100% n=126; non-screeners 98% n=86). Many had concerns about the nature of the FS investigation (screeners 72% n=91; non-screeners 66% n=58). Twenty-eight people who did not wish to be interviewed returned written responses, and these data were analysed thematically in conjunction with interview data. No significant differences in responses to the items included on the sampling questionnaire were identified between those who agreed to be interviewed (n=104) and those who did not (n=110). There were demographic differences between the recruitment areas with 71% of respondents from the North East from areas within the two highest quintiles of indices of multiple deprivation in the UK compared with only 4% of those from the East of England. Table 1 summarises the numbers of responses received and numbers of participants interviewed (screeners n=20; non-screeners n=25). Online supplementary appendices 1 and 2 provide a summary of the beliefs and attitudes of those interviewed towards screening along with key facilitators or barriers to attendance at the screening appointment.

**Table 1:** Summary of responses

	Male (East)	Female (East)	Male (North East)	Female (North East)	Total
Total questionnaire responses	55	70	34	55	214
Qualitative data totals	10	27	11	25	73
Written qualitative comments (non-screeners)	2	11	4	11	28
In-depth interviews (non-screeners)	3	11	3	8	25
In-depth interviews (screeners)	5	5	4	6	20

10.1136/bmjopen-2016-012304.supp1 supplementary appendix

**Figure 1:**
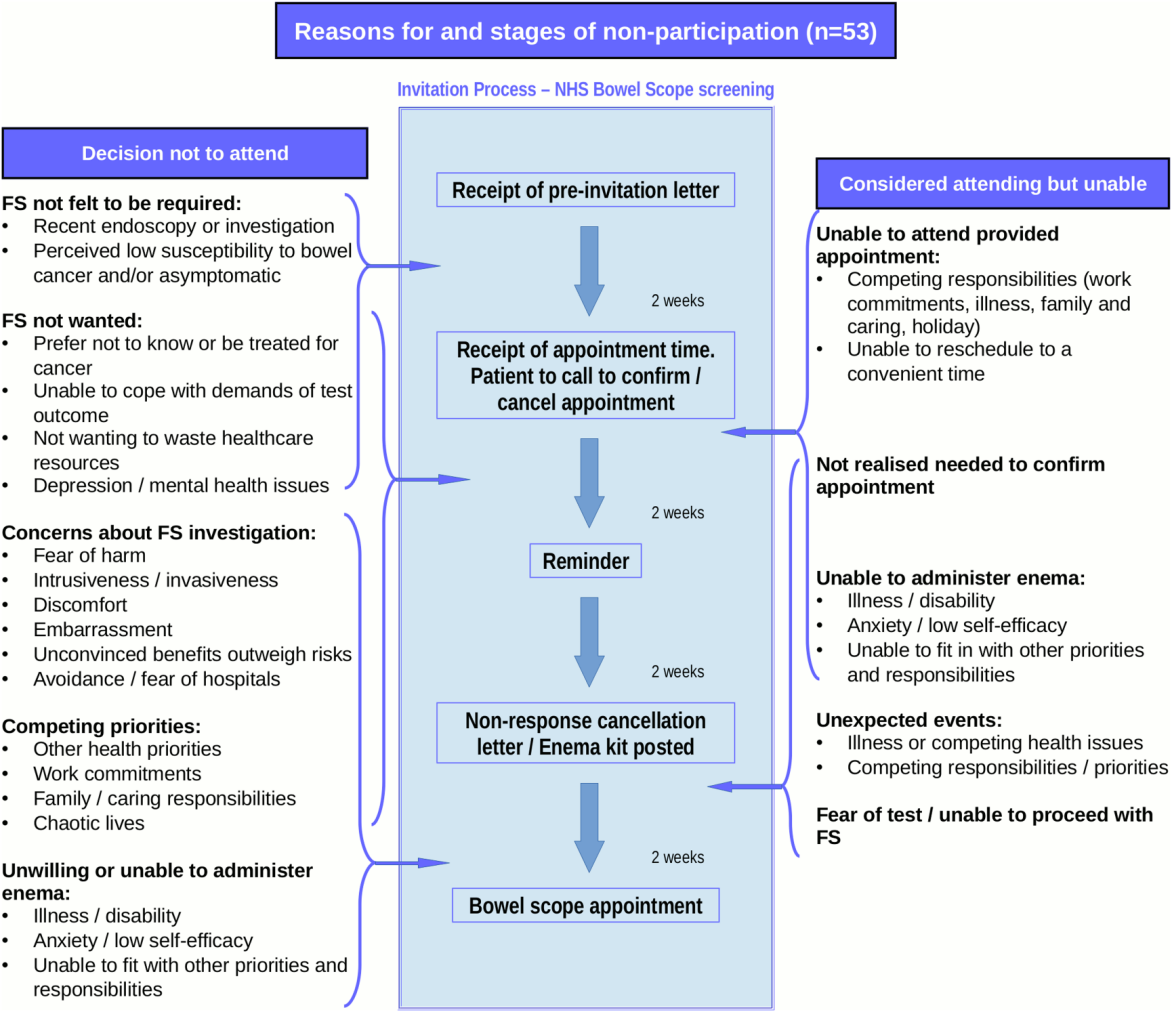
Summary of reasons for non-participation in flexible sigmoidoscopy (FS) screening in relation to screening invitation process.

Interview data and written comments demonstrated considerable heterogeneity in reported reasons for non-participation and multiple reasons were common. These are summarised in figure 1 in relation to the stages of the programme invitation, separating the reasons for unwillingness and inability to be screened.

The following section describes further in-depth exploration of the interview data in relation to the contexts, decision-making processes and emotional reactions rooted within the reasons provided for non-participation. These centred primarily around the balancing of concerns in relation to the FS test and a potential cancer diagnosis, beliefs about the need for reassurance, conflicting priorities and practical issues in relation to appointment scheduling. We use case examples (see box 1) as well as interview quotations to illustrate the complexity of these processes and to highlight some typical experiences of non-screeners in relation to their non-participation.

Box 1 **Case examples**

Typical case examples—non-screeners

**Case example 1:** Female non-screener (D-61)—decision not to be screened made based on consideration of harms and benefits.

Eileen is a retired midwife/nurse who had lots of experience working within the NHS and dealing with screening-related issues. Her father died of bowel cancer years earlier, and she described how he suffered for many years before he died. She thought very carefully about her response to the screening invitation and spoke to family members who encouraged her to attend, before calling to cancel her appointment. She attends all other cancer screening programmes and feels that her decision-making process for Bowel scope was very different and more involved than for other types of screenings. “The main thing that struck me was the statistics, you know one in 300 might have cancer, I though well I'm not having that shoved up my arse frankly for the sake of that, you know they might perforate my bowel.” She believes that finding bowel cancer early does not necessarily mean that you won't die from it and is not convinced that the Bowel scope programme “can be remotely cost effective”.

**Case example 2:** Male non-screener (D-34)—believed it is a good thing to do, but unable to overcome concerns about nature of the investigation.

Brian lives in a shared flat. He was shocked at receiving the invitation and initially assumed it was related to ongoing medical investigations for cancer. Once he realised this was not the case, he still felt this was something he probably should do, as “they probably screen people for a reason.” He felt very anxious because of the intrusive nature of the test and he talked to his mother, daughter and friends at the pub about it. They all encouraged him to take part. He also looked up further information about the procedure on the internet. After a long time deliberating, he decided he would go ahead with the screening, “grudgingly, I was kind of just thinking I would have it done.” He had not realised that he needed to confirm his attendance and then received a letter saying it had been cancelled. “At that point I thought relief to be honest and I decided to just leave it.” He feels screening is a good thing, especially at his age, but had never considered bowel cancer before. His father has died of prostate cancer and Brian feels he would be more in need of prostate cancer screening. “If nothing else I did read about it and it's opened my eyes to bowel cancer, which I'd never thought about at all, so it probably did some good.” He would encourage others to take part, but no longer has any intention to himself.

**Case example 3:** Female non-screener (C-71)—Bowel scope screening is currently unnecessary.

Barbara is retired and lives with her husband in a rural village. Her invitation arrived just 6 weeks after she had undergone a sigmoidoscopy following a GP referral for loose stools and rectal bleeding. “I am very much of the opinion that people should be responsible for their own health but to actually have the NHS just sort of knocking on your door and saying we'd like you to test this out for your peace of mind. I found it very welcome.” She recently lost a close friend to bowel cancer and currently has two other friends undergoing treatment for bowel cancer. After contacting the help desk, she was advised FS was not required at the moment, but she intends to take up the offer of screening before her 60th birthday as she is aware from her friends that the signs can be easily missed and she still has concerns “because of how I am normally, it might be a little bit difficult for me to actually isolate a change that's abnormal.”

**Case example 4:** Female non-screener (D-44)—Desires to be screened but unable to attend.

Rose is a full-time carer for her father and disabled daughter. She has lost her mother and close relatives to bowel cancer and feels concerned about her risks. She had a screening colonoscopy 5 years earlier and initially believed this was a repeat appointment. When she realised everyone was being invited for screening, she still wanted to take part. She was unable to attend her allocated appointment time. She has called twice to reschedule, but is unable to make an appointment far enough in advance to fit in with her caring responsibilities. “I can only go on these certain dates and they said well we can't give you them dates because we can only go up to a fortnight or so many days. I says well I can't do it then and I was a bit annoyed about that… I rang back and they were filled up again, so I didn't bother.” After that “I just forgot, I've got that much going on, I just forgot, that's all.” She is still willing to undertake screening and thinks that she might try again, but is aware she may potentially receive a further surveillance appointment sometime in the future.

### Concerns and perceived risk: invasiveness, embarrassment and potential harm

A general lack of awareness regarding the Bowel scope programme due to the early stage of national implementation at the time of our study meant that most interviewees with no prior experience or knowledge of endoscopic procedures described having reacted to their screening invitation with surprise or shock. The decision not to participate in screening for some had been based on a careful consideration of the perceived risks and harms associated with the test (see case study 1). The anticipated ‘unpleasantness’ of the FS procedure associated with its ‘invasiveness’ and potential embarrassment for screeners and non-screeners alike could however result in strong emotional reactions to the invitation (see case study 2). The FS test was described by some women as more intrusive and embarrassing than breast or cervical cancer screening, which were more easily normalised as part of being a ‘woman’.

The information provided in the screening invitation relating to potential harm and, in particular, bowel puncture had caused additional concern and anxiety for some interviewees.

I read the bit which stuck in my head that it could puncture your bowel…and I thought oh right I'm not doing that… I was just too scared to have it done. (D-60 female non-screener)

The bowel preparation (enema) was also described as a barrier. These concerns often only became apparent after the initial decision to attend screening, as the implications associated with the bowel preparation were not always clearly understood until further down the invitation process when the kit and instructions arrived through the post:

The only reason I cancelled my appointment was because after speaking to an assistant I realised I had to apply the enema an hour before attendance,… I could not do this at work. I would also feel worried about driving to the hospital after applying an enema! (C-4 female non-screener)

### Consideration of outcomes: perceived need for reassurance and likelihood of a potential cancer diagnosis

The anticipated unpleasantness of the procedures was often outweighed by strong beliefs about the personal need for reassurance offered by screening. It was the strength of perceived need for reassurance, rather than positive beliefs about the value of screening per se, that was more likely to be related to a rapid and/or firm decision to attend despite concerns about the procedure. A commonly reported need to put up with the inconvenience, embarrassment and unpleasantness of the test was evident within the accounts of screeners and the non-screeners who had wished to take part. However, the need for reassurance could override even intense anxiety about the procedure.

Panic. I didn't fancy this thing in my bum, but you've got to do it.… I've got to do this for my peace of mind. (C-39 male screener)

Similarly, a strong, and potentially legitimate, perceived lack of need (eg, due to a recent endoscopic investigation, see case study 3) was more likely to result in a firm non-screening decision. While the majority of interviewees, whether they had attended or not, held positive attitudes towards population-based cancer screening in principle and acknowledged the importance of early diagnosis, decision-making was more likely to be based on assessments of their own personal perceived susceptibility to bowel cancer. For some people, these beliefs were related to healthy behaviour choices or the presence of symptoms:

I do not feel at risk of bowel disease because I am not a heavy drinker, I hardly ever take pills and I have been vegetarian for 25 years and have an excellent diet and fitness regime, But I still think it's a great idea to offer this screening to people 55+. (D-63 female non-screener)

Personal experience of any type of cancer, either their own or of close others, seemed to heighten sensitivity to the need for reassurance and the importance of ‘catching cancer early’ (see case study 4). On the other hand, having witnessed suffering of a loved one after a long period of cancer treatment or remission could enhance fatalistic attitudes towards cancer.

Sometimes all these treatments and nothing works, so I think I would just give in at the first hurdle… they (friends with bowel cancer) went through all that battle and nothing worked. (D-60 female non-screener)

A preference not to know the outcome of screening was also described by those with existing physical and mental health conditions, particularly when associated with a reluctance to undergo treatment or a perceived inability to cope with the demands of a cancer diagnosis. Although there were some exceptions, most respondents' accounts described their understanding of FS screening as a diagnostic tool rather than a preventive measure.

### Responsibilities

A common narrative in the interviews of screeners and non-screeners was their sense of responsibility to take advantage of the screening opportunity. For some this included being accountable to the wider society, particularly in respect of the use of public (NHS) funding and resources, reporting an awareness that screening and early cancer detection was more cost-effective than later treatment and a responsibility to maintain their own health. However, appropriate use of resources was also described as a reason not to attend the Bowel scope appointment.

I won't have treatment for cancer… So, you know, I just think I'm not wasting people's, the NHS's money or whatever, you know, I'm just not. (D-83 female non-screener)

### Competing priorities and chaotic lives

Fitting in a screening appointment could be problematic when people were living chaotic lives, perhaps in deprived circumstances, caring for ill or disabled children or parents, or were faced with conflicting demands such as ill health. Difficulties attending a screening appointment were exacerbated when there was a sense of not having any reserves left to deal with potentially negative outcomes, other more immediate health concerns, or there were practical issues administering the enema or getting to hospital. The experiences of re-arranging inconvenient appointment times varied by screening centres, but the appointment system was a common barrier to many of those who had wished to take part and were unable to (see case study 4). Employment was another commonly reported competing priority. While appointments were in the evening and at weekends, the need to request time off work to attend could be a major barrier, even for those who were positive about screening.

### Decision-making and future intention

Although some interviewees reached a quick and firm decision about screening, decision-making was often described as a dynamic process and was more difficult when dissonant beliefs were held about potential screening outcomes and the need for screening.

If they found something, how would I react to that? Well I might be better off not knowing. But in the back of my mind that's saying yeah but it's better to know early. (C-112 female non-screener)

In these instances, decisions were reached with more difficulty and could change more easily and more frequently.

Many times I say no, I'm not going to do it, I don't want to have that in my body…one minute I was going yes, other minute I was going no…it wasn't an easy decision! (C-39 male screener)

Seeking additional information and talking to others was also more likely in these instances. Many non-screeners reported that they would consider taking part in screening in the future or had since decided to take part in FOBT screening when offered after their sixtieth birthday.

## Discussion

This is the first qualitative study that we are aware of to explore the decision-making of people who have been invited to attend FS screening when offered as an organised population-based programme. Our findings demonstrate that FS screening offered within this context is generally valued and associated with positive attitudes in relation to the importance of early diagnosis of cancer. These attitudes are held by those who do not attend screening as well as those who do. Our in-depth exploration of the contexts, decision-making processes and emotional reactions rooted within the reasons provided for non-participation showed that these centre primarily around the balancing of concerns about the FS test and potential cancer diagnosis, beliefs about the personal need for screening and reassurance, conflicting priorities and practical issues in relation to appointment scheduling.

The potential of FS screening to remove precancerous polyps, thereby allowing cancer prevention as well as detection, was absent in many accounts from screeners and non-screeners. When mentioned, this aspect of the screening was rarely described as having had a major influence on their decision-making. Our findings, nevertheless demonstrate that non-participation in FS screening is not necessarily due to a lack of knowledge, unjustified concerns or the lack of intention to attend an appointment. Furthermore, some participants felt their decision not to be screened was a rational and informed choice within the context of their individual circumstances.

One of the strengths of our research is that we were able to compare a diverse range of accounts from screeners and non-screeners within and across research sites and screening centres, including from participants living in the most deprived areas within the UK. Although our recruitment strategy, based on experience from a previous study,^14^ allowed for anticipated difficulties in recruiting non-screeners, the overall response to our study invitation was low. We were successful, nonetheless, in purposively sampling a diverse group of participants in terms of their beliefs, decision-making and reasons for non-attendance. We continued interviewing until data saturation was reached that is, no new themes were emerging from additional accounts. Our recruitment methods were based on qualitative purposive sampling.^24^ Further quantitative research would be required to ascertain the frequency of the different identified influences on decision-making within the wider population. The strength of our findings, however, lies within the in-depth exploration of the range of processes and influences involved in screening behaviour that is provided by our analysis of the accounts of our participants.

Efforts were made to ensure that interviewees did not feel judged about their non-attendance or coerced into future screening decisions. Although a degree of post hoc rationalisation is possible, our impression was that participants provided open and honest accounts of their experiences. Our sample did not include enough respondents from ethnic minority groups to draw conclusions about more specific cultural influences,^26^ and transferability of our findings to other regions and screening programmes may be limited. Despite including a mix of men and women and people from areas of high and low deprivation, we were not able to identify any influences specific to gender or deprivation. Further focused analysis in this area may, however, be of benefit, particularly as some of the barriers we identified including caring responsibilities, work practices and health benefits which are often gendered or socioculturally determined.

Our study complements the findings of a previous qualitative study of non-participation nested within in the UK FS Trial,^20^ as we were able to include and compare accounts of those with some intention to take part in FS screening but who were unable to, those for whom screening was not necessary and those who had attended screening. In contrast to their findings that practical barriers do not play a major role in screening uptake, we found that barriers such as inability to attend the screening appointment do play a role and that these barriers have a greater influence on actual appointment attendance than the initial decision or intention to attend. An analysis of variation in uptake during the first 14 months of the Bowel scope screening programme^7^ identified that centre effects have an independent association with uptake along with deprivation and gender and that those offered out of hours appointments were more likely to attend screening. Our participants' experiences and satisfaction with re-arranging inconvenient appointment times differed between screening centres. For some people, needing to request time off work to attend an appointment was seen to be embarrassing and was not always an appropriate option. Our study was completed in the early stages of the roll out of the Bowel scope programme when population and screening centre coverage was limited. Our findings indicate, however, that facilitation of uptake and satisfaction can be maximised by ensuring the flexibility of the appointment scheduling processes and accommodating those who would like to attend but are unable to when initially invited. Non-screeners who were undecided or unable to attend their initial invitation may benefit from an additional reminder at a later date. The integration of reminders into the screening programme has more recently also been shown by others to potentially be a feasible option worthy of further research.^27^ A more in-depth quantitative analysis on screening uptake including data on how many people confirm, reschedule or cancel their appointment would be beneficial to ascertain the portion of non-screeners who may benefit from improved flexibility or a safety netting approach to appointment rescheduling. Implications on programme delivery would also need to be considered. At present, Bowel scope clinic lists are ‘overbooked’ to accommodate non-attenders; a challenge for the programme is to balance service efficiency against capacity to maximise uptake while maintaining satisfaction with the appointment scheduling process and accommodating those who would like to attend but were unable to when initially invited.

Our findings confirm the importance of the influence of the perceived burden of the FS test, identified by others.^8^ ^12^ ^20^ ^28^ These concerns were evident across most participants, however, comparisons of accounts between screeners and non-screeners showed that when there is a strong perceived need for reassurance about potential cancer presence, concerns about the invasiveness of the test and other practical barriers were more readily overcome. The concept of ‘perceived need for reassurance’, as described in our analysis, reflects an appraisal response to the activation of emotional reactions triggered by the screening invitation, specifically in relation to beliefs about personal risks and circumstances. Our findings suggest that it is the strength of this perceived need rather than its presence that seems to be a crucial motivational driver of screening behaviour. It is directly influenced by beliefs about perceived personal susceptibility or vulnerability to cancer, coping style preferences, emotions and outcome expectancies (including fear and anxieties about the test itself, a potential cancer diagnosis and treatment), beliefs about screening and the sociocultural context. ‘Perceived need for reassurance’ therefore provides a useful lens through which to understand the decision-making process, as it allows for the involvement of a combination of existing sociopsychological constructs from health behaviour theories that have been used to explain and predict screening uptake, such as the extended health belief model.^29^ Crucially, ‘perceived need for reassurance’ also allows for the influence of emotion on behaviour and can be seen as being inextricably tied to the appraisal and coping processes that take place when faced with a health threat.^30^ The role of emotions and coping strategies in CRC screening behaviour has been previously reported. A questionnaire study of a subsample of the UK FS trial participants, for example, concluded that finding thoughts about cancer uncomfortable is associated with lower screening uptake and that different aspects of cancer fear can facilitate and inhibit screening intention and behaviour in different ways.^22^ The accounts from our participants illustrate and contextualise the strong emotions that can result from receiving a screening invitation associated with concerns not only about the screening process, but also with a potential cancer diagnosis and outcome expectancies associated with treatment beliefs and, for a small minority, general anxiety associated with hospital attendance. Our analysis also, therefore, complements findings by Oster *et al*^31^ that people's decisions to undergo CRC screening vary according to their degree of ambivalence towards finding out their cancer status and concurs with Palmer *et al*^32^ who propose that people do not wish to know the outcome of screening when they view treatment as ‘futile and unpleasant. Anticipated regret, the decision to take action to avoid experiencing unpleasant emotions associated with not having acted in a particular way, is a more cognitive-based emotional influence on screening uptake that has been found to have a complex relationship with CRC screening behaviour in a recent intervention study on FOBT screening,^13^ and this is reflected within our participants' accounts as an influence on their motivation to be screened.

The reported relationship between the lack of abdominal symptoms and lower uptake of CRC screening^8^ ^28^ can also be explained within the context of a lower perceived need for reassurance. Perceived susceptibility or vulnerability to a particular illness is an important element of many theories used to explain health behaviours such as screening.^18^ ^20^ When illness beliefs are associated with a lower perceived susceptibility to cancer, the motivation to be screened may not be strong enough to overcome any associated concerns. Within our participants' accounts, this was linked to healthy lifestyle choices as well as symptoms.

Our analysis demonstrates how decision-making processes in relation to FS screening are firmly situated within, and influenced by, the wider sociocultural context of people's lives, particularly in relation to their previous experiences with, and/or family history of, cancer. In the UK, uptake of CRC screening^11^ ^23^ and more specifically for FS^4^ ^7^ ^33^ is lower in areas of higher deprivation. Further research is needed to explore the mediating effects of factors associated with higher deprivation such as existing physical and mental health problems (that may affect the desire and/or ability to attend the screening appointment), the lack of desire for potential treatment and lower flexibility in employment leave. Difficulties assessing sociocultural norms around FS screening behaviour were commonly alluded to in our data, particularly for those with no prior experience or knowledge of endoscopic procedures. As the Bowel scope programme extends and awareness increases, ‘normalisation’ of FS screening may help to improve uptake^32^ and positive experiences of the screening procedures will be important in this regard. The importance of information on how other people deal with particular situations has been highlighted by others,^34^ and some participants suggested that knowing about the screening experience of others would have been helpful.

Cancer screening is often described in terms of a responsible or moral choice.^10^ ^14^ ^35^ Our findings demonstrate that the moral responsibility to ‘catch cancer early’ is a common and often dominant narrative. Even when present, however, these beliefs do not necessarily result in screening uptake and in some instances, non-participation in screening was also be explained in terms of moral choices and responsibilities, particularly within the context of wasting limited publically funded healthcare resources. Finally, our analysis highlights the need to acknowledge the dynamic nature of decision-making and screening intention within future research. Intention is often measured and reported as a relatively stable factor, however, our findings support a more variable stability of intention which should be taken into account when interpreting research findings in this area.

## Conclusions

In-depth exploration of the contexts, decision-making processes and emotional reactions rooted within the reasons provided for non-participation highlight the need to recognise the heterogeneity of non-screeners, particularly in relation to whether they are unwilling or unable to attend screening. Findings can inform the development and evaluation of targeted interventions and help to understand how psychosocial, provider and healthcare delivery factors interact to influence screening behaviour.
