# Supplementary material for: Concerns, perceived need and competing priorities: a qualitative exploration of decision-making and non-participation in a population-based flexible sigmoidoscopy screening programme to prevent colorectal cancer
Source: BMJ Open. 2016 Nov 11;6(11):e012304. doi: 10.1136/bmjopen-2016-012304 (PMC5129085; doi:10.1136/bmjopen-2016-012304)
Supplement: supplementary appendix [file bmjopen-2016-012304supp_appendix.pdf]

Appendix 1 – Interview participant attributes (Non-screeners N=25)

(Strongly agree = ++; agree= +' disagree= - ; strongly disagree = --; x=missing or n/a)

Sex	MDI rank (1-5) 5= most deprived	bowel cancer screening is important	I have concerning bowel symptoms	I was concerned about test	I was worried about what they might find	I am unable to have test for medical reasons	Previous cancer diagnosis	Cancer concern/susceptibility	Main reported reason for non-screening	Would consider in future?
**F**	X	++	+	+	+	+		Family history cancer (bowel). Not very concerned as recently tested after presenting with symptoms.	Endoscopy 3 months earlier, didn't want to waste resources	✓
**F**	2	+	-	++	-	-		Grandfather had cancer (bowel). Some implied concern believes takes a long time to develop	Embarrassment about test	✓
**M**	2	+	-	++	++	-		Lost friends to cancer but no more concerned with possibility of bowel cancer than any other illness	Attended but unable to proceed to test due to fear about potential damage caused by scoping	✓ Only if sedated
**F**	3	**x**	**x**	**x**	**x**	**x**		No specific concern. Has many other more prominent health problems	Not realised needed to confirm appointment	✓
**F**	2	++	+	--	--	--		Lost 1close friend to cancer (bowel), 1 had a diagnosis (bowel) but is fine and 1 is in terminal stages (bowel). High cancer concern and has current symptoms. Screening would offer reassurance.	Recent endoscopy after bowel change and bleeding	✓
**F**	x	**x**	**x**	**x**	**x**	**x**		No specific concern	Hadn't realised needed to call to confirm	✓
**F**	3	++	--	+	+	--		No specific concern	First appointment cancelled by screening centre. Appointment rebooked but clashed with hip operation	✓
**M**	1	**x**	**x**	**x**	**x**	**x**		No specific concern	Recently had a colonoscopy. Phoned and advised screening not needed. Would prefer to let someone else have appointment.	✓
**F**	5	++	--	+	+	--		Family history of cancer (breast).	Unable to get time off work to attend	✓

Sex	MDI rank (1-5) 5= most deprived	bowel cancer screening is important	I have concerning bowel symptoms	I was concerned about test	I was worried about what they might find	I am unable to have test for medical reasons	Previous cancer diagnosis	Cancer concern/susceptibility	Main reported reason for non-screening	Would consider in future?
**F**	3 (+)	++	-	-	+	--		Strong family history (bowel) and high cancer concern	Unable to attend appointment due to caring responsibilities	✓
**F**	2	++	-	+	-	-		No specific concern	Chronic illness affects ability to get to hospital	✓
**F**	1	+	--	+	-	-		No specific concern	Potential risk of test. Undergoing other medical treatment.	✓
**M**	2	++	-	-	-	--		No specific concern	Life too chaotic following marriage breakdown	✓
**F**	1	++	--	--	--	--		Lost father to suspected cancer. Hadn't considered own susceptibility. Lives healthily.	All seemed too messy and inconvenient. Couldn't administer the enema at work	**?**
**F**	1	++	-	--	**x**	-		No specific concern	Panic about hospitals. Last scope 2 years ago was painful. Couldn't administer own enema. No opportunities for sedation.	**?**
**F**	3	++	+	--	--	--		2 friends with cancer and good recovery. Feels less at risk as eats healthily.	Unable to administer the enema on own due to MS and appointment not convenient, No chances to reschedule.	**?**
**F**	2	+	-	+	-	-		No specific concern	Does not feel unhealthy	**?**
**F**	2	+	--	+	-	--		No concern. Feels safe in own self care and awareness	Friends reported bad experience. Has ongoing health issues relating to cirrhosis and does not wish to risk complications due to caring responsibilities for mother.	**X**
**F**	1	++	-	--	**x**	-		No specific concern	Fear of hospitals. Complicated medical history and sigmoidoscopy 2 years earlier.	**X**

Sex	MDI rank (1-5) 5= most deprived	bowel cancer screening is important	I have concerning bowel symptoms	I was concerned about test	I was worried about what they might find	I am unable to have test for medical reasons	Previous cancer diagnosis	Cancer concern/susceptibility	Main reported reason for non-screening	Would consider in future?
**F**	5	+	-	+	+	-	✓	Lost mother to breast cancer. High concern.	Has other conflicting responsibilities and concerns about intrusiveness of test	**X**
**F**	2	+	(ongoing bowel sx**)**	+	-	-		Lost 1 friend recently and 1 currently in palliative care (both bowel ca). Currently on mind and has ongoing bowel symptoms	Main reason was the fear of harm/damage. Also belief that bowel cancer is hard to treat.	**X**
**F**	1	+	--	++	-	--		No specific concern	Not realising what test involves until enema arrived in post.	**X**
**F**	1	+	--	++	--	--		**Lost father to cancer (bowel ca).**	Not convinced by statistics provided or that benefits outweigh invasiveness of test. Feels treatment can prolong suffering. Attends other cancer screening.	**X**
**M**	4	+	-	+	+	--		Lost both parents (mother OG ca). High cancer concern.	Initially, appointment during holiday. Then decided he does not want to know if cancer.	**X**
**M**	3 (+)	+	--	+	-	--		Father (prostate ca) but low bowel cancer concern.	Unpleasantness of FSIG	**X**

Appendix 2 – Interview participant attributes (Screeners N=20)

(Strongly agree = ++; agree= +' disagree= - ; strongly disagree = --; X=missing or n/a)

Sex	MDI rank (1-5) 5= most deprived	bowel cancer screening is important	I have concerning bowel symptoms	I was concerned about test	I was worried about what they might find	Previous cancer diagnosis	Cancer concern/susceptibility	Main reported reason for screening
**M**	1	++	--	+	+	✓	High cancer concern	Had cancer before and knows benefits of catching things early
**M**	1	++	++	+	++		Family history of bowel cancer (father and grandfather). High concern and recently presented to GP with symptoms.	To put mind at rest
**M**	2	+	-	+	+		Cousin diagnosed with cancer (bowel) and friend. No particular concern but experienced bleeding from haemorrhoids.	Wants peace of mind
**M**	1	++	+	+	+		Brother in law and friend with bowel cancer with different outcomes.	Importance of finding early/reassurance.
**M**	1	++	--	+	+		No specific concerns, feels well.	Would prefer to know than not. Seemed sensible approach.
**M**	1	++	-	+	-	✓	High awareness due to own cancer and concerned may have a problem and not know.	Own cancer and awareness of the importance of catching early.
**F**	2	++	+	+	+		Uncle with cancer (bowel). High concern due to family history and own symptoms	Wanted reassurance due to uncle’s diagnosis and own symptoms.
**F**	1	++	+	+	-		High concern due to previous polyps.	Opportunity for reassurance due to previous polyps.
**F**	1	++	-	+	-	✓	Some concern.	Opportunity to catch anything early and better chances of survival.

**F**	2	++	-	+	++		Lost mother (bowel ca). Concerned about family history.	Concern family history and opportunity for reassurance.
**F**	2	++	-	++	+		Mother diagnosed with cancer (bowel) and father (bladder). High cancer concern	Didn't want to miss opportunity for reassurance and importance of catching it early. Encouraging people to go for screening is part of her job.
**F**	3	++	+	+	++	✓	Feels susceptible to any cancer and aware of previous rectal bleeding	For peace of mind. Has had previous rectal bleeding.
**F**	5	++	+	+	+		No specific concern.	Aware of bowel symptoms, and prefer to find out and catch it early.
**M**	1	++	-	+	+		No specific concern	Importance of catching early. Responsible use of resources. Benefits outweigh the unpleasantness.
**M**	3	++	--	-	-		Lost father to cancer (bowel). High cancer concern and experienced symptoms	Positive about opportunity. Wanted reassurance as feels susceptible.
**F**	3	++	+	++	-		Aunt diagnosed cancer (bowel) and mother (bladder). Lost friend to cancer (breast). Concerned about susceptibility and symptoms. Does not feel at any higher risk than others, can strike anyone.	Reassurance about ongoing bowel changes, experience of close relative with bowel cancer.
**F**	3	++	--	x	+		Lost brother to cancer (bowel). High cancer concern	Thought it was a recall for genetic screening. Wanted reassurance.
**M**	3	++	++	++	+		Mother and father in law died ca and friends at work. General cancer concern.	Wanted peace of mind. Prevention better than cure.
**M**	3	++	--	+	++	✓	No specific bowel cancer concern until screening invite, but ongoing cancer treatment.	Thought appointment was related to ongoing cancer investigations/treatment. Good to have reassurance
**F**	1	+	+	++	+		Lost 1 friend to cancer (breast) and another had a good outcome. Mother recently had precancerous polyp removed.	Conscious about bowel cancer, glad for reassurance and good opportunity.
